# Twin tubular pinch effect in curving confined flows

**DOI:** 10.1038/srep09765

**Published:** 2015-04-30

**Authors:** Liviu Clime, Keith J. Morton, Xuyen D. Hoa, Teodor Veres

**Affiliations:** 1 National Research Council of Canada 75 Boulevard de Mortagne, Boucherville (Quebec) J4B 6Y4, Canada; 2 Institut National de la Recherche Scientifique (INRS) 1650, boulevard Lionel-Boulet, Varennes (Québec) J3X 1S2, Canada

## Abstract

Colloidal suspensions of buoyancy neutral particles flowing in circular pipes focus into narrow distributions near the wall due to lateral migration effects associated with fluid inertia. In curving flows, these distributions are altered by Dean currents and the interplay between Reynolds and Dean numbers is used to predict equilibrium positions. Here, we propose a new description of inertial lateral migration in curving flows that expands current understanding of both focusing dynamics and equilibrium distributions. We find that at low Reynolds numbers, the ratio *δ* between lateral inertial migration and Dean forces scales simply with the particle radius, coil curvature and pipe radius as 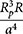. A critical value *δ_c_* = 0.148 of this parameter is identified along with two related inertial focusing mechanisms. In the regime below *δ_c_*, coined subcritical, Dean forces generate permanently circulating, twinned annuli, each with intricate equilibrium particle distributions including eyes and trailing arms. At *δ* > *δ_c_* (supercritical regime) inertial lateral migration forces are dominant and particles focus to a single stable equilibrium position.

Buoyancy neutral particles flowing in circular pipes show a tubular pinch effect where particles are focused in a reproducible manner into a narrow annulus near the wall^1^. The lateral force responsible for migrating particles toward the wall is associated with the inertia of the fluid^2^,^3^,^4^,^5^ and the effect scales up and down to any particle size or tube diameter as long as the continuum hypothesis for the fluid flow remains valid. In the years following the discovery of the tubular pinch effect, the majority of related literature was concerned with theoretical aspects of lateral migration of particles. In the limit of small Reynolds numbers (compared to 1), analytical models for the lift force in unbounded^3^,^4^ or bounded^2^,^6^,^7^ linear shear flows have proven useful in capturing most essential features of the effect. Moreover, numerical simulations based on lattice Boltzmann mesoscopic approaches have further validated the equilibrium focusing positions of particles up to Reynolds numbers of about 1000^8^. Accurate numerical models for the drag, lift and torque for non-spherical particles in linear shear flows have also been presented recently^9^. Interesting changes in the topology of the equilibrium positions are observed in curving flows where lateral migration effect is combined with drag forces induced by Dean flows^10^. Accurate control of particle focal positions at extremely high flow rates by controlling the viscoelastic properties of the fluid carrier has been demonstrated as well^11^.

In recent years, the inertial focusing effect is increasingly being used in microfluidic devices with rectangular cross-section channels for high throughput separation and filtration applications. In rectangular geometries, suspended particles migrate to at least four stable equilibrium positions along the channel periphery. These equilibrium positions can be collapsed into just two streams in high aspect ratio cross-section channels^12^,^13^ and into one single stream by employing curvilinear channels that induce Dean flows^14^,^15^. Several authors^15^,^16^,^17^,^18^,^19^ have presented extensive experimental results on the later effect and tried to capture observed behavior into simple design rules and diagrams based on dimensionless quantities such as the channel Reynolds number, the particle Reynolds number and the Dean's number. However, the particle dynamics in an inertial migration process as well as accurate theoretical models and related physical quantities to describe this dynamics remain challenging.

Here we investigate both theoretically and experimentally the inertial lateral migration effect in curving flows and highlight several unique features in the spatial distributions of focused particles in a regime where Dean flows dominate over inertial lateral migration effects. One of the more interesting aspects of this regime is the development of two annular distributions of particles that are entrained by the two Dean vortices in a continuously counterrotating motion to form a twin (double) tubular pinch effect. We also investigate the opposite regime where lateral migration forces dominate over Dean drag and highlight fundamental differences as well as transition mechanisms between these two regimes. Field forces acting on particles are described in terms of stable, unstable and saddle equilibrium points within the framework of a simple analytical model while trajectories and topology of particle clouds are investigated numerically. Confocal microscopy measurements of fast flowing particles are presented in order to corroborate these theoretical findings.

## Results

### Analytical model for inertial lateral migration velocity in curving flows

**Figure 1:**
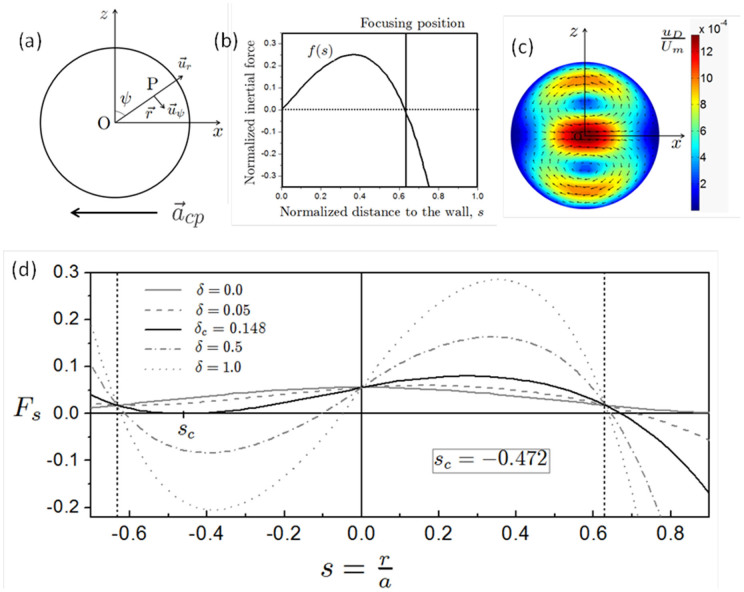
Analytical model for inertial focusing effect in curved pipes. a) Schematic representation of the transversal cross-section of the pipe in both Cartesian *xOz* and polar coordinates. By convention the center of curvature of the coil is in the negative direction of *Ox* axis as indicated by the centripetal acceleration vector 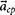; b) Graphical representation of the tabulated function *f*(*s*) for Re < 50 according to Shonberg and Hinch^21^. The focusing position corresponds to the coordinate *s*_0_ = 0.631; c) Contour fill plot and vector field of the Dean flows normalized to the axial flow velocity U_m_ according to Dean^10^ for Re = 10 and Dn = 1; d) Plots of *F*(*s*) along axis *Ox* at different values of parameter 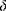. The critical value 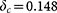 is highlighted with a solid continuous black line. The two vertical dashed lines at *s* = −0.631 and *s* = +0.631 highlight the position of the focusing points in the absence of the Dean flows. Center of curvature of the tube is at the left (in the region *x* < 0).

In a circular pipe of cross-section radius *a*, the velocity profile of an incompressible liquid flowing in the laminar regime is well approximated by the parabolic profile 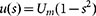. *U_m_* is the maximal velocity while 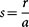 is the normalized cross-sectional position of the particle (Fig. 1a). The liquid flow includes a relatively small volume concentration of particles, typically less than 3% such that the tubular pinch effect is not inhibited^20^. In pipes coiled into a circle of radius *R*, Dean flows are generated by the fluid inertia and the velocity field develops a two-vortex transverse flow (perpendicular to the pipe central line) defined by^10^
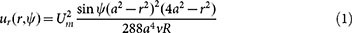
and
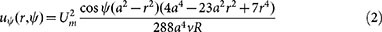
where *v* is the kinematic viscosity of the liquid, 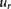 and 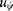 are the radial and circumferential components of the Dean velocity field, respectively. To quantitatively describe the Dean flow, we retain from Eq. (1) the maximum Dean velocity 
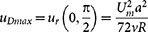
 and observe that the ratio of Dean to axial flow maximum velocities scales as
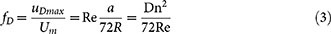
where Re and Dn denote respectively the channel Reynolds number 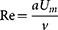 and the Dean number 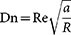. To maintain the physical validity of the analytical model given in Eqs. (1) and (2), Dean^10^ suggests that the number 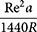 (that is 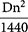) be kept as small as possible, usually less than unity. For a circular channel of radius 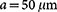 forming a coil of curvature radius *R* = 1 cm this condition limits the flow to Re = 536.

For Reynolds numbers up to 100, Schonberg and Hinch^21^ demonstrated that in laminar flows in pipes of cross-section radius *a*, buoyancy neutral spherical particles develop an overall lateral migration velocity that can be written as
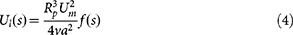
with *R_p_* the radius of the particle and *U_m_* the maximum velocity of the liquid flow. *f*(*s*) is a dimensionless inertial lift parameter that for a given Reynolds number, depends on the normalized cross-sectional position *s* only. At low Reynolds numbers, *f*(*s*) has been already tabulated by Schonberg and Hinch^21^ by considering full parabolic Poiseuille flows and wall effects. As shown in Fig. 1b, this function has two zeros: one is at the origin *s* = 0, that is the center of the pipe, and another at *s*_0_ = 0.631. While the first zero is trivial and simply means that the particles moving on the central line of the pipe do not experience any inertial focusing effect, the second one relates to the well-known tubular pinch effect and indicates stable equilibrium positions for particles^1^,^21^ on an annulus of radius 0.631*a*.

By combining inertial lateral migration in Eq. (4) with Dean flow effects in Eqs. (1) and (2), we obtain the full velocity field for particles flowing in a curved pipe. The radial and angular components of this field can be rewritten as
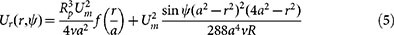
and
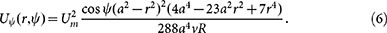
By analysing the topology of these two vector fields (one radial and the other as shown in Fig. 1c) we conclude that the only regions where the resultant field can vanish are either on the axis *Ox* (in the pipe central plane) or at *x* < 0 and *r < s_0_a* where radial position vector 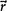 is tangent to the Dean streamlines (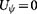). For all other off-axis regions (above and below *Ox* axis), the two velocity fields have different directions and nonzero components at all points with two exceptions: (i) the centers of the two Dean vortices (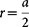 and 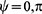) where the Dean drag velocity is zero and (ii) the circle of radius *r* = *s*_0_*a* where lateral migration force vanishes. At these points, the particles will experience only one of the two forces but the overall resultant force will still be non-zero, thus particles at these points cannot be considered in equilibrium. We will restrict then our analysis to the axis *Ox*, by considering 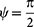 in Eq. (5). The fact that the off-axis points in the region *x* < 0 and *r < s_0_a* are not equilibrium points either will be demonstrated later on by full numerical simulations.

The only non-zero scalar component of the resultant velocity vector on the axis *Ox* becomes then
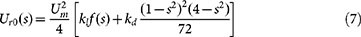
where 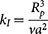 and 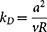 are two parameters related to the inertial migration and Dean drag velocities, respectively. By employing the notation
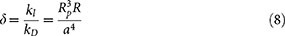
Eq. (7) becomes
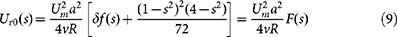
where
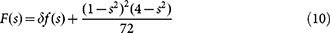
was also employed. Of interest in the topology of this function are the roots of the equation *F*(*s*) = 0 that indicate potential equilibrium positions of particles in the channel. It is interesting to observe in Eq. (10) that 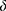 is the only parameter responsible for changes in the topology of the function *F*(*s*) and its related zeros. It is also interesting to observe from Eq. (8) that this parameter is purely geometric, as it does not rely on the physical properties of the liquid or the associated flow Reynolds number. This is limited to flows where theoretical models expressed by Eqs. (1–2) and (4) remain valid, that is at low Reynolds numbers. However, since Dean's model in Eqs. (1) and (2) is valid up to Re = 536 while *f*(*s*) chosen for this study (Fig. 1b) imposes Re < 50, it is rather the inertial lateral migration term that limits the model to relatively slow flows. Consequently, if we want to extend the present model to 50 < Re < 536, a new function *f*(*s*) appropriate for the range of interest has to be employed^21^ and all above formalism remains unchanged.

### Solutions of the equation *F*(*s*) = 0 and related equilibrium points

**Figure 2:**
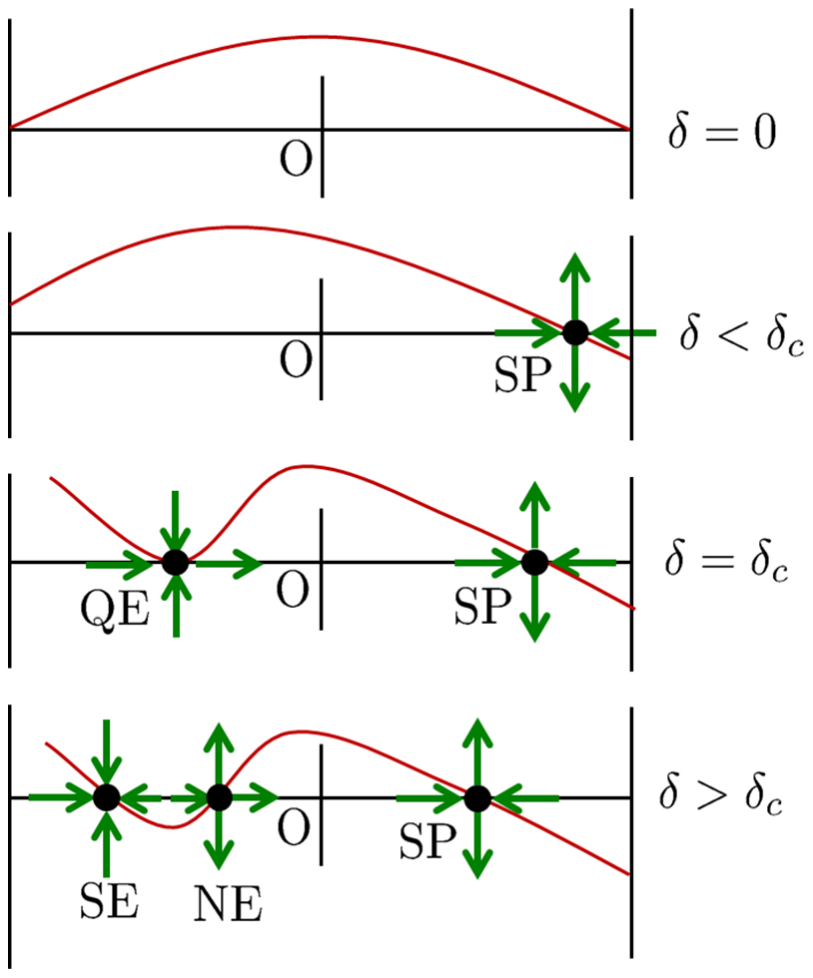
Topology and solutions of the equation *F*(*s*) = 0. Depending on the values of the dimensionless parameter 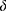, four types of feature equilibrium points are illustrated: the saddle point (SP),the quasi-equilibrium (QE) point in the critical regime, the non-equilibrium (NE) and stable equilibrium (SE) points in the supercritical regime. Centripetal acceleration vector has the same orientation as in Fig. 1 (center of curvature to the left).

We investigate in the following the topology of the function *F*(*s*) at some critical values of the parameter 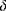. While exact graphical representations of this function for different values of 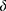 are presented in Fig. 1d, some additional drawings in Fig. 2 are used to show qualitatively the orientation of the drag forces at each point of interest and identify the type of equilibrium for each configuration.

At 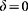, the profile of the function *F*(*s*) is symmetrical with respect to the ordinate axis (Fig. 1d) and has two zeros at *s* = −1 and *s = +1*. At these points the particles are however at rest since no-slip boundary conditions are imposed by the Poiseuille flow profile. Since 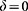 just removes the inertial contribution in *F*(*s*) this means that in the absence of inertial lateral migration effect equilibrium positions along the axis of interest (*O*x) other than the extreme points *s* = ¸1 are not possible.

As the parameter 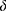 is increased (by increasing the size of the particles for example), *F*(*s*) becomes slightly asymmetric and the zero in the region *s* > 0 (far side of the coil) begins to move away from the wall (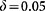 in Fig. 1d). However, as depicted in Fig. 2 (curve 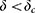) this zero cannot be a stable equilibrium position for the particles since the off-axis (vertical) velocity field is divergent. The particles will leave this point at the smallest perturbation (induced by Brownian motion for example). Consequently, this particular point corresponds to a saddle point (SP).

Interestingly, while the saddle point SP continue the shift toward the origin with increasing 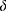, at 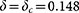 the function *F*(*s*) becomes tangent to the abscissa at the point *s_c_* = −0.472 (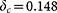 in Fig. 1d). The off-axis velocities are now converging toward this equilibrium point (curve 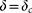 in Fig. 2) but on the abscissa particles still experience a divergent velocity field since *F*(*s*) is positive on both sides of this critical point. Consequently, this will be an unstable quasi-equlibrium point (QE) as well. Particles at this point will be at rest for a relatively short period of time since they are pushed toward the center of the pipe at the smallest external perturbation such as from Brownian motion or interparticle collisions. As discussed later, this critical value 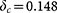 of the parameter 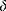 will play a major role in our analysis as it is responsible for the separation of the observed behavior across three regimes, each with completely different particle dynamics and focusing patterns, regimes that we coin as subcritical for 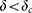, supercritical for 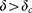 and critical for 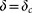.

Further on, when 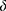 gets larger than the critical value 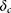 we observe a splitting of the point QE into two zeros, both in the region *s* < 0: one relatively close to the origin and another close to the inner wall, near the default focusing point *s* = −*s*_0_ = −0.631. Again the point near the center (O) will not be stable since the velocity field is divergent in all directions (Fig. 2), thus this point will correspond to a non-equilibrium (NE) state. In contrast, for the root near the inner wall, we observe that all the conditions for static equilibrium are fulfilled: the velocity fields in both on-axis and off-axis directions converge toward this point. In this case particles will settle into a stable equilibrium (SE) position. This is in fact the most commonly reported result in the literature where very stable and narrow streams of particles can be obtained. We observe however, that the position of this stable position changes slightly with 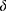 in the sense that as 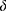 gets larger, the equilibrium position gets closer to the wall having as limit the critical point *s*_0_ = −0.631, where the focusing in the absence of any Dean flows is supposed to occur (Fig. 1d). Since the parameter 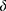 is related to the size of the particles through Eq. (8), this simply implies that larger particles are biased toward *s* = *s*_0_ while smaller particles will prefer to focus nearer to the point *s_c_*. An example of a separation application based on this effect was already demonstrated by Kuntaegowdanahalli *et* *al.*^18^ It is interesting to observe that all the particles will be distributed in the interval [*s*_0_, *s_c_*] that is between −0.631 and −0.472. Consequently, according to the present theoretical framework, the use of this effect for particle size separation is limited to a rather narrow window of just 16% of the pipe radius, that is about the same order of magnitude as the diameter of the particles responsible for an observable inertial focusing effect in that pipe. According to Kuntaegowdanahalli *et* *al.*^18^, the critical particle size in inertial focusing experiments has to be *R_p_* ≥ 0.07*a* that is 14% of the pipe cross-sectional radius. It is however possible that this interval be slightly extended in channels with high aspect ratio rectangular cross-sections but this is not investigated in the present study.

### Flow rate and channel length critical parameters

The discussion so far focuses on the topology of the dimensionless functions *f*(*s*) and *F*(*s*) which describe the distribution of resultant drag force and related equilibrium points in the cross-sectional area of the channel. In this respect, the discussion is limited to the position of these final equilibrium points without regard to the time required for particles to reach them. According to Eq. (9) the resultant velocity of the particles scales as 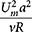. For large values of this factor, particles will move faster toward equilibrium positions while at smaller values, the particles will focus slower. In experimental work, controlling this parameter is very important since the pipes (channels) will always have a finite length in which the focusing is expected to occur. Consequently, while dynamics of the particles in the cross-sectional area of the pipe will give an insight into the way particles attain equilibrium positions, the total necessary length of pipe has to be considered accordingly.

By convention, we consider a particle traveling a length 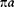 along a Dean vortex at a velocity 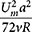, that is the maximal velocity of the Dean flows at the center of the pipe (*s* = 0). In the same amount of time, the flow in the pipe will advance axially along the pipe center line with the quantity


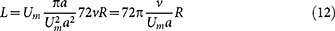
Dividing this length *L* by the circumference of the coil, we obtain the number of turns required for the flow to complete the rotation of the particle in the Dean flow:


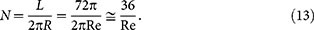
This result is rather interesting since the number of turns necessary for a Dean off-axis displacement is fixed by the Reynolds number regardless the particle and the pipe configuration parameters. From a practical point of view, it is important then to have a Reynolds number of at least a few tens in order to have particles focused after a few turns only.

### Particle dynamics in inertial curving flows

To account now for the dynamics of the particles toward the equilibrium positions as well as the topology of stable and unstable equilibrium configurations, we have developed a simple numerical model based on the superposition of the two (lateral inertial migration and Dean) velocity fields as shown in Eq. (5) and (6). The resultant total field vector 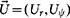 resulting from these two equations is coupled to a diffusive velocity field 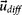 and used to solve the differential equation of motion


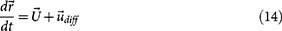
by using a fourth-order Runge-Kutta integration scheme^22^ and a random walk diffusion model^23^ (see Methods for more details on the numerical model). Although the particles used in this study are rather large (the smallest particles are 2 *µ*m in diameter with associated diffusion constant 
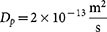
), we maintain the term 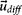 in Eq. (14) in order to give the simulation some degree of realism: without this term particles are trapped at saddle and non-equilibrium points indefinitely.

The simulation starts with a number of few tens of particles randomly distributed at the inlet section of the pipe. The positions of these particles are then evolved according to Eq. (14) and axial projections of these positions in the pipe cross-sectional plane are plotted at each time step. Particle configurations as well as their migration toward equilibrium are investigated in the three regimes: (i) subcritical (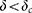) when the function *F*(*s*) has a single zero point (SP); (ii) the region around 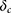 – critical – where the critical point QE is about to split into the stable (SE) and the non-equilibrium (NE) points and (iii) supercritical (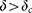) where the critical point SE approaches the limit *s*_0_ = −0.631 of the inertial focusing in the classical tubular pinch effect.

### Subcritical regime (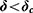)

**Figure 3:**
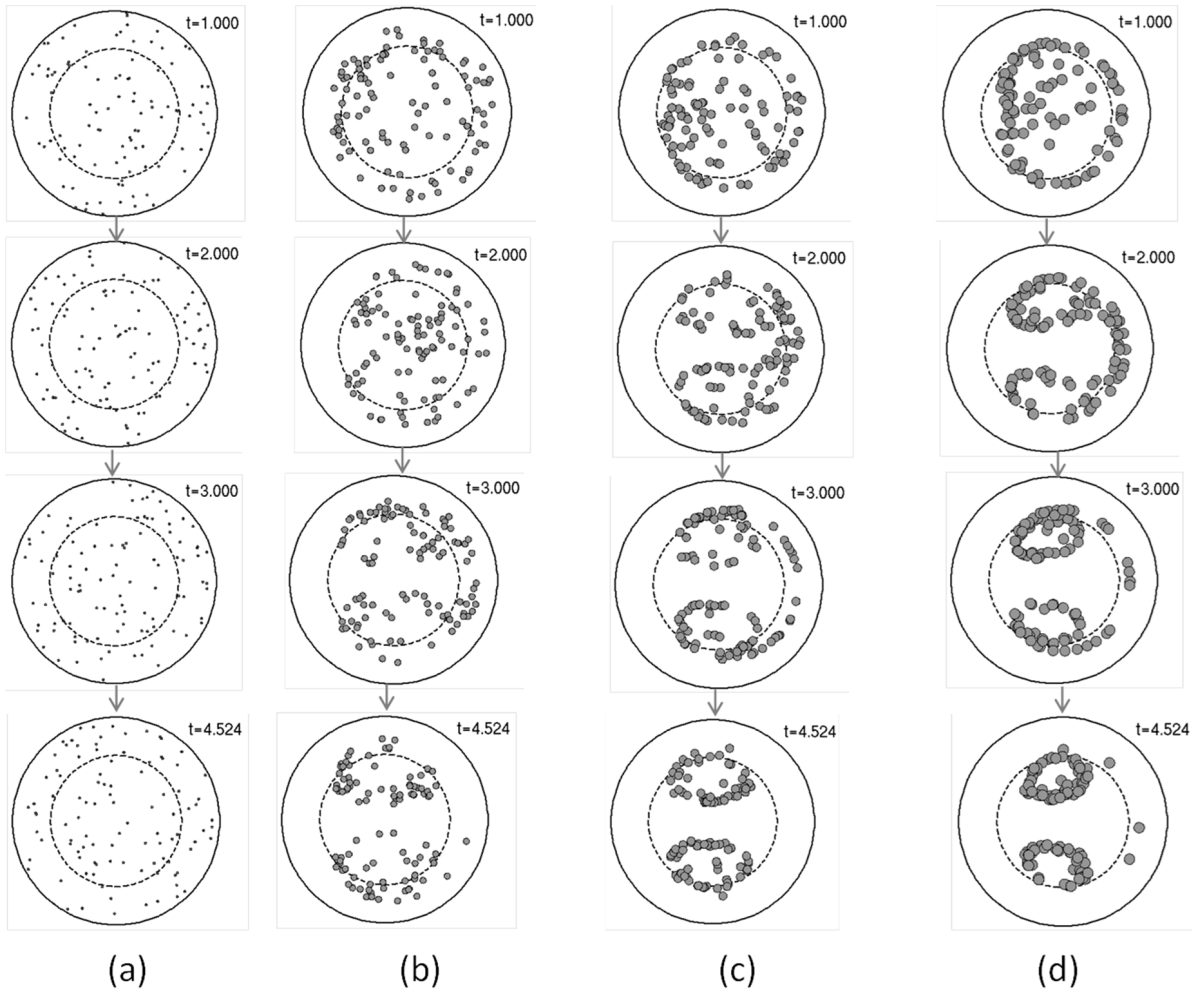
Inertial focusing of small particles. Numerical simulations of inertial focusing in curving confined flows in the subcritical regime at: a) 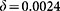; b) 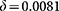; c) 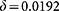 and d) 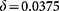. These values correspond to particles with diameters of respectively 2, 3, 4 and 5 *μ*m in a 100 *μ*m diameter channel and Re = 16. Computer animations of the particle cloud time evolution are presented in the supplementary video files movie_3a.mp4, movie_3b.mp4, movie_3c.mp4 and movie_3d.mp4, respectively.

**Figure 4:**
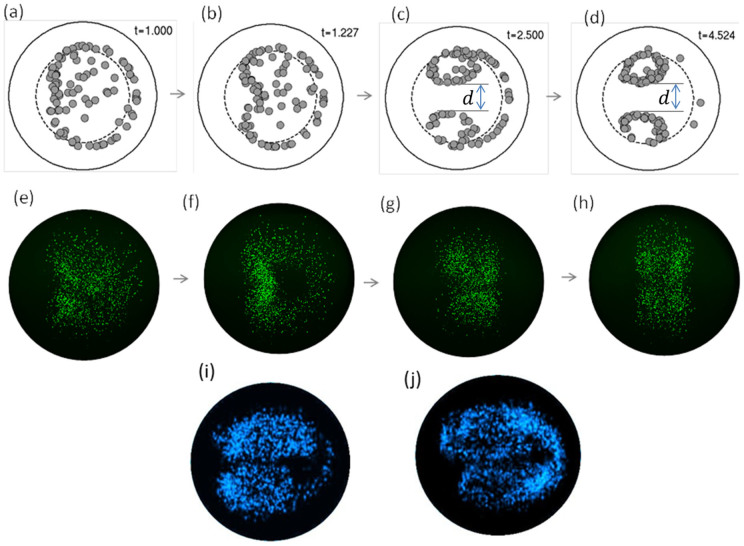
Inertial focusing in curving channels at 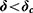. (a–d) Numerical simulations of the time evolution of particle radial positions in the subcritical regime at Re = 16 (100 *μ*l/min) and 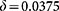 (5 *μ*m diameter particles in 100 *μ*m diameter tube); e–h) Experimental confirmation by confocal microscopy measurements at Re = 16 (e–h) and same value of parameter 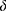; i–j) Smaller particles (2 *μ*m diameter) flowing faster at Re = 22 (150 *μ*l/min) and Re = 30 (200 *μ*l/min), respectively. Size of particles are to scale in all simulations but not in experimental images.

Numerical solutions of Eq. (14) at 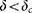 and Re = 16 indicate the formation of two twin annuli of particles that are symmetric about the coiling plane (Fig. 3) and entrained into two counterrotating vortices by the Dean currents. Very small particles do not show this behavior since they remain mostly unfocused and simply follow the Dean flows while they are advancing axially in the channel (Fig. 3a). As the size of the particles increases, the two annuli become better defined while the center of each annulus becomes clearer with a net vertical separation between them (Fig. 3b–d). Confocal measurements of buoyancy neutral fluorescent beads flowing in curved capillary tubes at 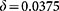 are compared to theoretical simulations (Fig. 4a–d) and confirm this behavior (Fig. 4e–h) by clearly showing the formation of two distinct focusing annuli and the development of a separating gap (*d*) at the coil symmetry plane (Fig. 4c–d and Fig. 4g–h). Details about the confocal measurement setup are presented in the Methods section and in the Supplementary Information material. However, to clearly visualize experimentally the two annuli and the trail of particles – as indicated by the simulation – the channel Reynolds number had to be increased and the size of the particles diminished slightly (Fig. 4i–j). This indicates that the quantity *N* (number of turns) as defined by Eq. (13) slightly underestimates the number of loops needed for complete and stable inertial focusing. This can originate in the approximations employed for the characteristic length 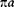 and associated fluid velocity to derive Eq. (12) and could be addressed by considering different phenomenological parameters better related to the length and the average speed of particles on these contours.

Color and contrast-enhanced cross-sectional scatter plots in the experimental figures represent confocal laser hit-points of fast flowing particles in curved capillary tubes. It is important to note that the points here do not correspond to actual particles centroids, but the scatter patterns are rather an approximation of the particle probability distribution field. For this reason, data points in the confocal images look rather scattered and pattern features corresponding to inertial focusing are more clearly defined in small particle experiments (Fig. 4i–j) rather than for large ones (Fig. 4e–h).

**Figure 5:**
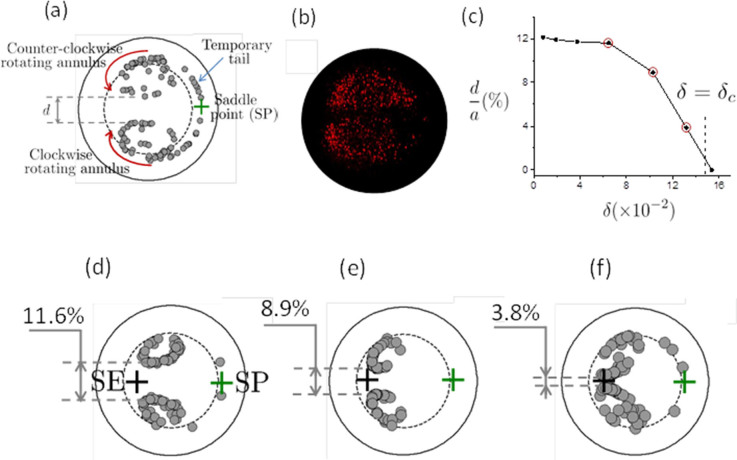
Inertial focusing in curving channels at 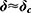. a) Numerical simulation of particle distributions in the subcritical regime highlighting some relevant features such as the twin counterrotating vortices, the saddle point, the gap *d* between the two annuli and the temporary tail; b) experimental confocal microscopy image of a mix of 3 *μ*m (red) particles in a pipe of 100 *μ*m diameter (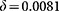); c) dependence of the gap between the two annuli *d* on the parameter 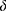; d–f) the configuration of the two annuli for the three points on the curve 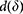 highlighted in figure c). Particle sizes are to scale in all simulations but not in experimental images.

Since the parameter *t* in the simulation corresponds to the number of complete turns covered by particles, the relatively large values for the time t > 4 employed in simulation compared to *N* = 2.26 as given by Eq. (13) show that the observed patterns are stable and will not continue to evolve regardless the total length (number of coils) of the pipe. While the formation of the two twin annuli is a relatively fast process (after two turns only), entraining all particles into this twin annuli pattern is a very long process. The reasons for these two distinct time scales is the presence to the saddle point (SP) at s > 0 where particles can reside for a long period of time until small perturbations (such as those induced by Brownian motion) move them away from these points. As a direct consequence, particles will form tails (trains) centered on the saddle point (Fig. 5a) that will deplete slowly until all particles are irreversibly trapped in the rotating twin annuli. This behavior is also confirmed by confocal measurements at 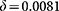 (Fig. 5b) where the twin annuli pattern and the tail generated by the saddle point are observed.

### Critical regime (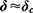)

**Figure 6:**
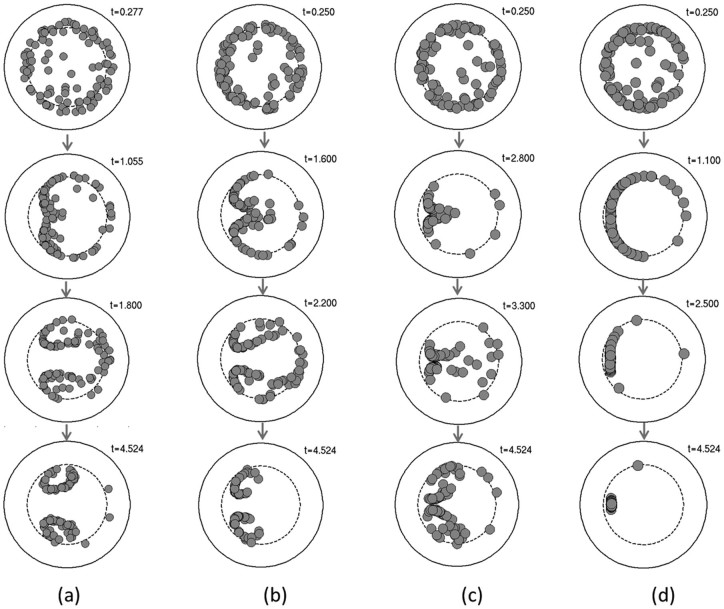
Inertial focusing of large particles. Numerical simulations of inertial focusing in curving confined flows at the transition between subcritical and critical regimes: a) 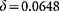; b) 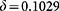; c) 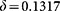 and d) 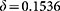. These values correspond to particles with diameters of respectively 6, 7, 7.6 and 8 *μ*m in a 100 *μ*m diameter channel and Re = 16. Computer animations of the particle cloud time evolution are presented in the supplementary video files movie_6a.mp4, movie_6b.mp4, movie_6c.mp4 and movie_6d.mp4, respectively.

An interesting evolution of the topology of the two annuli pattern is observed when 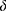 approaches the critical value 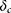. From the numerical simulations at small values of 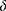 in the previous section we conclude that the two annuli are almost centered between the stable equilibrium (SE) and saddle (SP) points. As 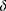 is increased, the annuli move toward the stable equilibrium point (SE) and approach each other by diminishing the separation gap *d*. This reduction is initially rather modest with increasing 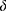 (Fig. 5c) but rapidly closes down above 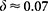 (Fig. 5d–e) such that near the critical value 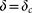 the gap vanishes completely and the two annuli touch each other (Fig. 5f). The size of these annuli is gradually reduced as well in the radial direction making the transition towards the regime where particles are focused into a single stream near the stable equilibrium point SE. More numerical simulation results that support the above description of the transition toward the critical regime are presented in Fig. 6a–d and related supplementary material.

### Supercritical regime (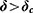)

**Figure 7:**
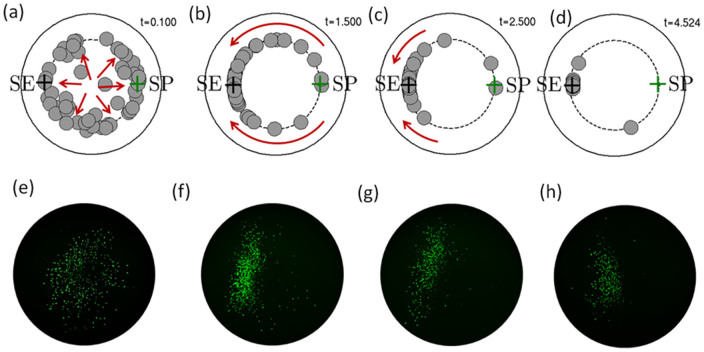
Inertial focusing in curving channels at 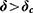. Numerical simulations (a–d) and experimental confocal measurements (e–f) of time evolution of inertial focusing at 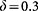 for 10 mm diameter particles. Size of particles are to scale in all simulations but not in experimental images.

Beyond the critical regime the two vortices have practically no radial extent and are collapsed to a single large annulus (regular tubular pinch effect) where particles are pushed toward the stable equilibrium point SE by the Dean currents.This regime is the most frequently reported in the literature and corresponds in our theoretical framework to the case where inertial migration forces are dominant. Focusing in this regime is a two-step process, confirmed by both numerical simulations and confocal microscopy experiments (Fig. 7). First, the particles are sent to the focusing points on a ring corresponding roughly to *s* = *s*_0_ (the regular tubular pinch effect). This process is fast in the sense that the particles focus to an annulus within the first turn (as shown in Fig. 7a) then Dean flows then start to displace particles on this ring towards the stable equilibrium point SE (Fig. 7b). Contrary to the subcritical regime where particles were continuously cycled on and off this focusing ring (by the formation of the twin counterrotating annuli), the inertial migration forces in the supercritical regime are strong enough to impede particles from leaving their equilibrium positions along the ring. As the flow advances in the coiled pipe, more and more particles are packed around the stable equilibrium point while the saddle point is gradually depleted (Fig. 7c and d).

It is notable that even after more than three complete turns of the coiled piped there are still off-equilibrium floating particles (Fig. 7d). This is mainly due to the influence of the saddle point (SP). These theoretical conclusions are again well supported by experimental confocal images where the annulus corresponding to the tubular pinch effect is well formed (Fig. 7e) while the evolution toward the stable equilibrium positions (Fig. 7f–h) is very similar to that obtained by numerical modeling. This is also the reason that for practical applications we need to design coils with an actual number of turns larger than the parameter *N* (from Eq. 13) to ensure that particles trapped near the saddle point SP will have enough time to leave that point and migrate toward a stable equilibrium position.

## Discussion

The theoretical framework proposed in this paper provides two important parameters for predicting the inertial focusing behavior of particles in curved coils: 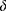 and *N*. While the first is used to distinguish between subcritical (twin tubular pinch effect) and supercritical (regular tubular pinch effect) regimes, the second can be thought of as an estimate of the tube length (number of coils) needed to achieve an observable effect. Interestingly, the number *N* depends only on the Reynolds number and becomes equal to unity at 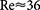. From a more practical point of view, particles of any size with 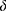 higher or slightly below the critical value 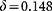 will give an observable effect within a reasonable number of turns as long as a Reynolds number in the order *O*(10) is guaranteed. This raises some difficulties in applications with small particles since the required Reynolds numbers can be difficult to achieve as they imply narrower channels and large applied driving pressures.

Parameter 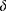 as defined by Eq. (8) does not replace the well-known inertial focusing condition *R_p_* ≥ 0.07*a* for the tubular pinch effect in straight pipes^12^. In other words, the divergence 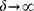 for coils of zero curvature simply means that the particles will be fully in the inertial focusing regime since 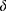 is larger than 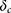. However, what is observed in this work is the formation of interesting topologies of stable particle distributions even when the inertial focusing condition *R_p_* ≥ 0.07*a* is not fulfilled – that is when particles are smaller than the critical size claimed by this condition. The experimental results shown in Fig. 4 highlight the dynamics of the two subcritical annuli where the particles are smaller than stated by this condition. For the other regime (supercritical) Gossett and Di Carlo have published state diagrams and design rules^17^ to be used for single and multiple turn curved channels. However, with our analysis we offer a more complete picture of the actual mechanism of focusing not only in the supercritical but in the subcritical regime as well that cannot be captured by these simple diagrams.

The fact that particles can be focused to well defined stable equilibrium configurations despite the fact the condition *R_p_* ≥ 0.07*a* is not fulfilled opens the path to interesting separation applications where, by designing appropriate outlet bifurcations and pumping protocols, inertial focusing of small species can be achieved in larger and shorter inertial focusing channels. We believe that the new proposed theoretical framework has the potential to support sample preparation applications in areas such as food safety inspection, environmental screening or clinical diagnostics where isolation and concentration of small microbial organisms (e.g., bacteria, parasites or fungi) is of primary concern.

## Methods

### Numerical model

The equation (14) is solved in the cross-section plane of the pipe that is by taking into account only the components of the velocity that are perpendicular to the flow. In this way the problem is reduced to a system of two scalar differential equations and solved with a fourth-order Runge-Kutta algorithm^22^. In order to account for diffusion processes, a random walk model has been implemented^23^ by considering in the right hand term of Eq. (14) the quantity where 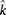 is a randomly oriented unit vector. 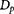 and *t* here are the equivalent diffusion constant of the particles in the respective liquid according to the Stokes-Einstein equation^24^ and the time step of the simulation, respectively. Initial condition consists of few hundred particles randomly distributed at the pipe at the inlet and flowing at the velocity of the liquid on the respective streamline. Positions of the particles are evolved in the cross-sectional plane according to Eq. (14) regardless their axial position and by neglecting any mutual interactions between particles. Images representing the positions of the particles with respect to the pipe walls are generated at each time step and assembled in computer simulation movies that accompany Figs. 3 and 6.

### Microchannel fabrication

The microfluidic device is constructed from 100 µm (ID) HPFA capillaries (IDEX Health & Science, Illinois, US), mounted on a glass carrier in a three loop configuration each of 1.5 cm radius (as illustrated in Supplementary Fig. 1). The input and output capillary ends are connected to a computer controlled syringe pumps (PHD 2000 Harvard Apparatus, MA, US) and a waste reservoir, respectively. The capillary is then encased in a layer of polydimethylsiloxane (PDMS) forming a 2 mm thick slab. The PDMS minimize the optical diffraction distortions by matching closely the refractive index of the cylindrical HPFA capillary and the glass substrate. Particles used in microfluidic experiments consist of solutions of Fluoro-Max fluorescence microbeads (Thermo Scientific, MA, US). Solutions are prepared at 0.01% (W/V) of 2 µm, 3 µm and 5 µm in distilled water, doped with Rhodamine.

### Confocal microscopy measurements

Microbead tracking is performed on a NIKON Ti-Eclipse C2 Laser scanning confocal microscope. The laser raster scans a narrow window (100 µm) in the longitudinal direction of the capillary and across the full width of the channel. Z-stack is obtained from scan at step of 250 nm covering 150 µm, the full cross-section of the capillary tube. FITC 488 nm and DSRED 561 nm lasers are used to excite the microbead and the background fluorescence from the Rhodamine doped solution. The cross-sectional images of the capillary are then aggregated and sum along the scanned longitudinal axis to form a composite image of the tracked microbial in the capillary tube. These images provide a map of positions at which the laser intersects a flowing particles thus individual points on the image do not necessarily represent a particles. These can rather be associated with a probability distribution of particles over the cross-section of the capillary with higher density corresponding to higher probability of presence of particle flow. A schematic representation of the experimental setup as well as some examples of primary 3D confocal images are given in the Supplementary Material.

## Supplementary Material

Supplementary Information Supplementary Information

Supplementary Information Supplementary Movie3a

Supplementary Information Supplementary Movie3b

Supplementary Information Supplementary Movie3c

Supplementary Information Supplementary Movie3d

Supplementary Information Supplementary Movie6a

Supplementary Information Supplementary Movie6b

Supplementary Information Supplementary Movie6c

Supplementary Information Supplementary Movie6d
