# Supplementary material for: Twin tubular pinch effect in curving confined flows
Source: Sci Rep. 2015 Apr 30;5:9765. doi: 10.1038/srep09765 (PMC5386211; doi:10.1038/srep09765)
Supplement: Supplementary Information [file srep09765-s1.pdf]

# Supplementary Information
## Twin tubular pinch effect in curving confined flows

L. Clime, K.J. Morton, X. D. Hoa and T. Veres

### Experimental setup

A confocal laser scanning microscope (Ti-E C2+ with Laser Scanning Confocal, Nikon, Canada) is used to measure the distribution of particles in the micro-sized pipe channel. Three-dimensional images are formed from a laser raster scan of a window covering the full width of the channel (yz plane) and 100 µm (x-axis) pipe length. 2D projections along the coordinate axes are reconstructed using a computer software (NIS Element, Nikon, Canada) from the acquired fluorescence images. The coiled microfluidic device channel is formed using a plastic tube with circular cross-section of 150 $\mu$m inner diameter (HPFA+ 360 µm x 150 µm, IDEX Health &Science, US) coiled along a micro-machined groove in a rigid plastic substrate (COP Zeonor 1060R, Zeonex, US) forming a three-loop circuit as shown below in Supplementary Fig. 1A. To minimize optical distortions produced by refractive index mismatch at the fiber-air interface, a rectangular windows is cut in the substrate covering the regions of interest (as indicated in Supplementary Fig. 1A) and Polydimethylsiloxane (PDMS) silicon polymer is casted over the area. This removes the polymer-air curved optical dioptre with a flat PDMS-air interface. The inlet is connected to a syringe pump (PHD Ultra Syringe Pump, Harvard Apparatus, US) and solutions of fluorescent particles of diameters between 2 and 10 $\mu$m are injected through the device at Reynolds numbers of about 20. Examples of confocal measurements for a fiber of 150 $\mu$m diameter and 10 $\mu$m fluorescent particles are shown in Supplementary Figs. 1 B, C and D.

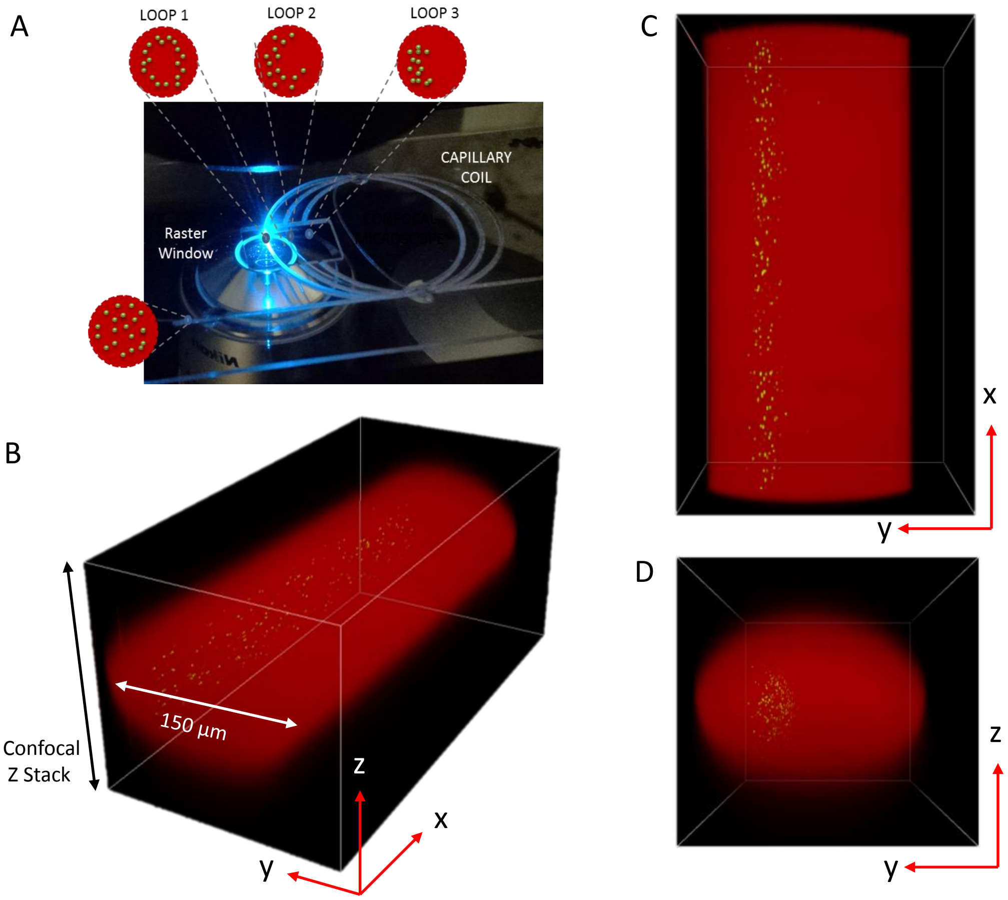


**Supplementary Figure 1** | Schematics of the confocal measurement setup (A) and some selected experimental results on the inertial focusing of 10 µm diameter beads flowing at Re=20 in a circular pipe of 10 µm diameter: B) 3-D view; C) XY view and D) YZ view. Center of curvature of the coils in Figs. B, C and D is to the left (in the direction of Y axis).
